# PB.24: Is digital breast tomosynthesis helpful within a symptomatic one-stop breast clinic for characterisation of subtle findings?

**DOI:** 10.1186/bcr3524

**Published:** 2013-11-08

**Authors:** GJ Bansal, P Young, K Lim, Z Rees

**Affiliations:** 1 University Hospital of Llandough, UK

## Introduction

The study investigated the accuracy of two-view full-field digital (2D) mammograms (FFDM) by comparing its performance with two-view digital breast tomosynthesis (3D) plus FFDM combination in a symptomatic setting.

## Methods

A multi-case multi-reader study was conducted involving four imagers of varying experience. A total of 109 lesions from 103 patients who attended symptomatic breast clinics during a 7-month period between March 2012 and September 2012 were retrospectively read. All patients who had subtle signs on 2D images were retrospectively double read in a free response study and the findings recorded. The performance quality of the methods was evaluated using receiver operating characteristic (ROC) curves. Reader agreement (*k *values) was calculated and the area under curve (AUC) was compared between 2D imaging and 2D+3D imaging, regarding both readers and cases as random events.

## Results

There was more inter-reader agreement between the two readers with 2D+3D combination (*k *= 0.391) compared with 2D images alone (*k *= 0.153). In total, 85.19% of M3 mammograms on 2D imaging were changed to 11.01% M3 and 77.06% M2 on combination of FFDM and DBT. There was more correlation between the mammographic scores and the final result for combination mode compared with 2D images alone (*P *= 0.0001). ROC analysis revealed the AUC for the combination (0.898) was significantly greater than 2D mammograms alone (0.734; *P *= 0.009).

## Conclusion

DBT increases diagnostic sensitivity in a symptomatic setting and reduces the number of M3 mammograms, when used as an adjuvant to 2D images. Thus DBT has the potential of increasing work-flow efficiency in a symptomatic setting by reducing benign biopsies.

